# New thiazole derivative as a potential anticancer and topoisomerase II inhibitor

**DOI:** 10.1038/s41598-024-81294-1

**Published:** 2025-01-03

**Authors:** Mayada I. Shosha, Fawzia Z. El-Ablack, Entsar A. Saad

**Affiliations:** Chemistry Department, Faculty of Science, Damietta University, Damietta, New-Damietta, 34517 Egypt

**Keywords:** Isoindole-1,3-dione, Topoisomerase II, CT-DNA-binding, Molecular docking, Rhodanine, Hybrids, 1,3-Dioxoisoindole, Biochemistry, Cancer

## Abstract

To shed light on the significance of thiazole derivatives in the advancement of cancer medication and to contribute to therapeutic innovation, we have designed the synthesis and antiproliferative activity investigation of 5-(1,3-dioxoisoindolin-2-yl)-7-(4-nitrophenyl)-2-thioxo-3,7-dihydro-2H-pyrano[2,3-d] thiazole-6-carbonitrile, the structure of thiazole derivative was confirmed by spectroscopic techniques UV, IR and NMR. The cytotoxic activity (in vitro) of the new hybrid synthesized compound on five human cancer cell lines; human liver hepatocellular carcinoma (HepG-2), colorectal carcinoma (HCT-116), ﻿breast adenocarcinoma (MCF-7), and epithelioid carcinoma (Hela), and a normal human lung fibroblast (WI-38) was studied using MTT assay. The compound exhibited a strong cytotoxicity effect against HepG-2 and MCF-7. The interaction of the newly synthesized compound with calf-thymus DNA (CT-DNA) was investigated at pH 7.2 by using UV–Vis absorption measurements, also, molecular docking was carried out to investigate the DNA binding affinity of the proposed compound with the prospective target, DNA (PDB ID: 1d12). Finally, molecular docking was carried out to examine the binding patterns with the prospective target, DNA-Topo II complex (PDB-code: 3QX3). Results indicated that the investigated compound strongly binds to CT-DNA via intercalative mode, and correlated with those obtained from molecular docking and in agreement with that of in vitro cytotoxicity activity.

**Supplementary Information:**

The online version contains supplementary material available at 10.1038/s41598-024-81294-1.

## Introduction

Cancer presents a major global public health challenge, remaining one of the most deadly diseases worldwide. Medicinal chemists are working hard to find new, targeted, safer, and powerful anticancer drugs^1–4^. The isoindole-1,3-dione pharmacophore has become a key area of focus in drug discovery and development for various purposes. This moiety has been identified as a key structure for designing novel drug candidates with multiple biological activities such as being androgen receptor antagonists^5^, anticancer^6^, antioxidants^7^, antiviral activities^8^, anticonvulsant^9^, antimicrobial^10^, hypoglycemic^11^, antihyperlipidemic^12^, anti-inflammatory^13^, anti-Alzheimer^14^, xanthine oxidase inhibitors, carbonic anhydrase inhibitors^15^, and antileishmanial against *Leishmania amazonensis* and *Leishmania braziliensis*^16^. Recently, several compounds with this structure have been developed and researched as inhibitors of SARS-CoV-2, effectively combating the spread of the COVID-19 pandemic^17^. The N-substituted isoindole-1,3(2H) dione scaffold has a distinctive structure important for its biological activity. It includes a hydrophobic aryl ring, a hydrogen bonding domain, electron-donor groups, and other architectural characteristics that depend on the substitution at the N atom. These substitutions may influence the biological activity^18^. Moreover, compounds containing the thiazole core structure have demonstrated noteworthy biological impacts, including anticancer^19–22^, antimicrobial^23^, antiviral^24^, antioxidant^25^, antiprotozoal^26^, antidiabetic^27^, anti-inflammatory^28^, anticonvulsant^29^, antipsychotic^30^, and anticholinesterase activities^31^.

Furthermore, several commonly used medicines contain a thiazole core, such as abafungin and ravuconazole for antifungal purposes, ritonavir for anti-HIV treatment, febuxostat for gout, nizatidine for ulcers, imidacloprid as an insecticide, myxothiazols and melithiazols as fungicides, fatostatin as a sterol regulatory element-binding proteins (SREBPs) inhibitor, tiazofurin, bleomycin, and dasatinib as anticancer drugs, fanetizole, meloxicam, and fentiazac as anti-inflammatory medications, sulfathiazole as an antimicrobial, and nitazoxanide as an antiparasitic agent, and penicillin as an antibiotic^31–34^.

Furthermore, 4H-pyrans are an important class of heterocycles containing oxygen, 4Hpyrans exhibit a variety of biological characteristics. Several natural products or pharmaceutical compounds have the 4H-pyran core, which is typically found as a component of 4H-chromene skeletons (4H-1-benzopyrans)^35,36^. The 4H-pyran ring compounds exhibit miscellaneous pharmacological features, such as diuretic, spasmolytic, anti-coagulant^37^, antimicrobial^38^, mutagenicity^39^, antitumor^40^, antiviral^41^, sex pheromone^42^), and antiproliferative^43^ properties.

DNA topoisomerases are unique enzymes that modulate the topological linkages between DNA strands through cleavage and relegation mechanisms, altering the number of superhelical turns^44^. On the basis of their mechanisms, topoisomerases can be classified into two major classes: type I and type II DNA topoisomerases. Type I DNA topoisomerases cleave one single-stranded DNA during each catalytic cycle. Type II topoisomerases break one double-stranded DNA strand, allowing another segment of duplex DNA to pass through the transitory breaking before resealing the broken strand to solve DNA knots and tangles. Type II topoisomerases are subdivided based on the structure-homology and the mechanism of action into type IIA and type IIB. Type IIA includes type IIα and type IIβ in humans^45^.

Topoisomerase II (Topo II) is a promising molecular target for anticancer agents, such as doxorubicin, amsacrine, mitoxantrone, and etoposide^46–48^. Topo II inhibitors are traditionally classified into two groups: catalytic inhibitors and Topo II poisons, based on their mode of action. Catalytic inhibitors work by disrupting the enzymatic activities of Topo II, which prevents the formation of the Topo II–DNA complex without increasing DNA cleavage. They can use interference with DNA linking, suppression of DNA cleavage, ATP hydrolysis, and linking to the ATP linking site for achieving their action. Topo II poisons destroy tumorigenic cells by enriching the amount of covalent Topo II–DNA complexes and inhibiting the rejoining of the cleaved DNA strands. This leads to the formation of harmful double-strand breaks in the DNA, which are toxic to the cells and ultimately result in programmed cell death (apoptosis). Depending on how they bind to DNA, Topo II poisons can be DNA-intercalating compounds.

Taking into account the significance of isoindoline-1,3-dione, and the pharmaceutical applicability of pyran and thiazole-containing drugs, and building on our previous work in creating bioactive 1,3-thiazoles^49–53^, our goal is to develop a new heterocyclic compound that combines these heterocycles and to investigate its potential anticancer effect, with the hope of achieving potent biological activity.

## Results and discussion

### Chemistry

Molecular hybridization is a strategy for designing new promising ligands. It involves recognizing pharmacophoric units in bioactive parent molecules and fusing them to create new hybrid architectures. These new designs maintain the preselected pharmacophoric characteristics of the original molecules^54,55^.

**Fig. 1:**
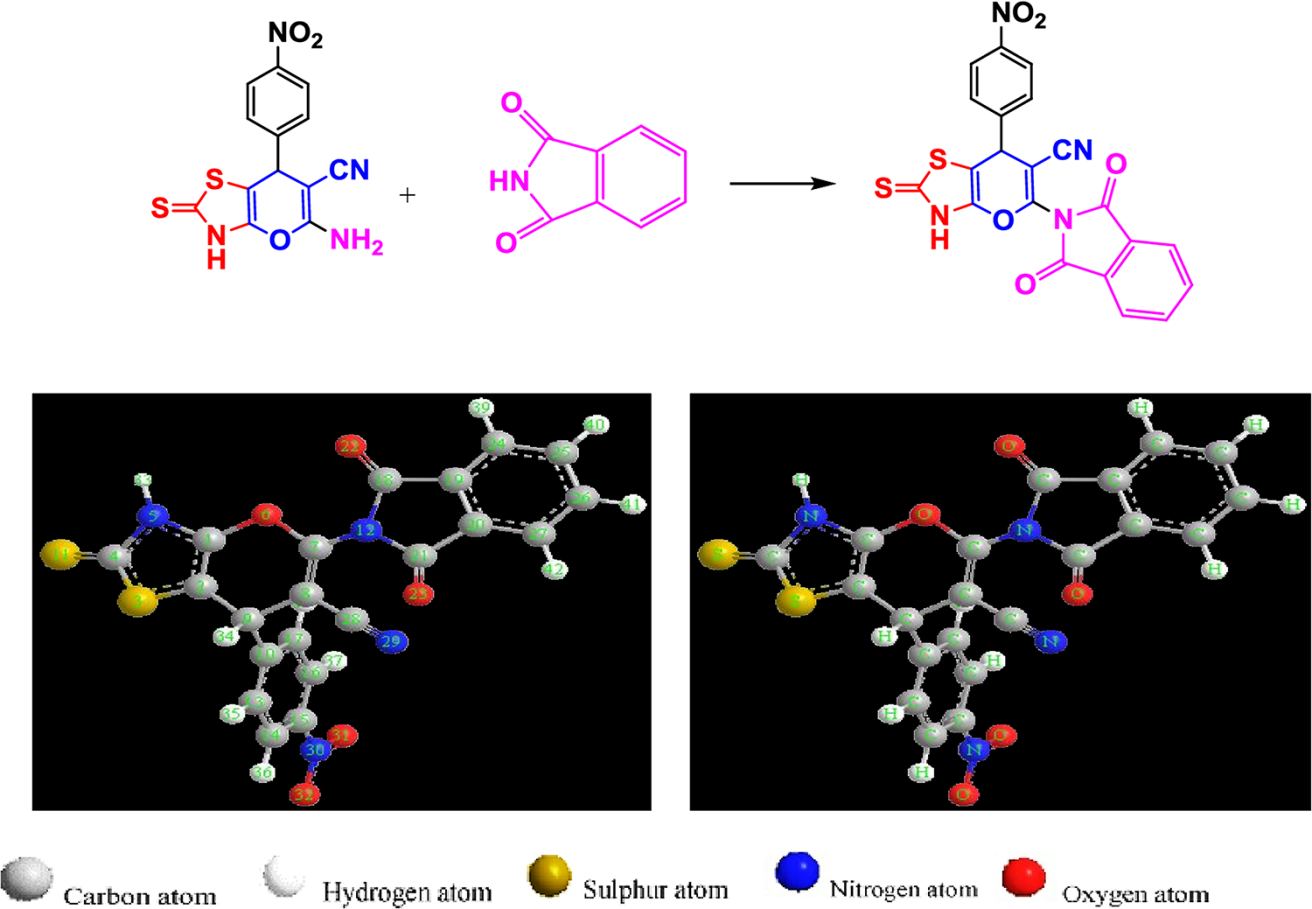
Synthesis of 5-(l,3-dioxoisoindolin-2-yl)-7-(4-nitrophenyl)-2-thioxo-3,7-dihydro-2H-pyrano[2,3-d]thiazole-6-carbonitrile (**Upper panel**) as well as geometry optimized structures of the synthesized compound with balls and cylinders rendering (**Lower panel**).

**Fig. 2:**
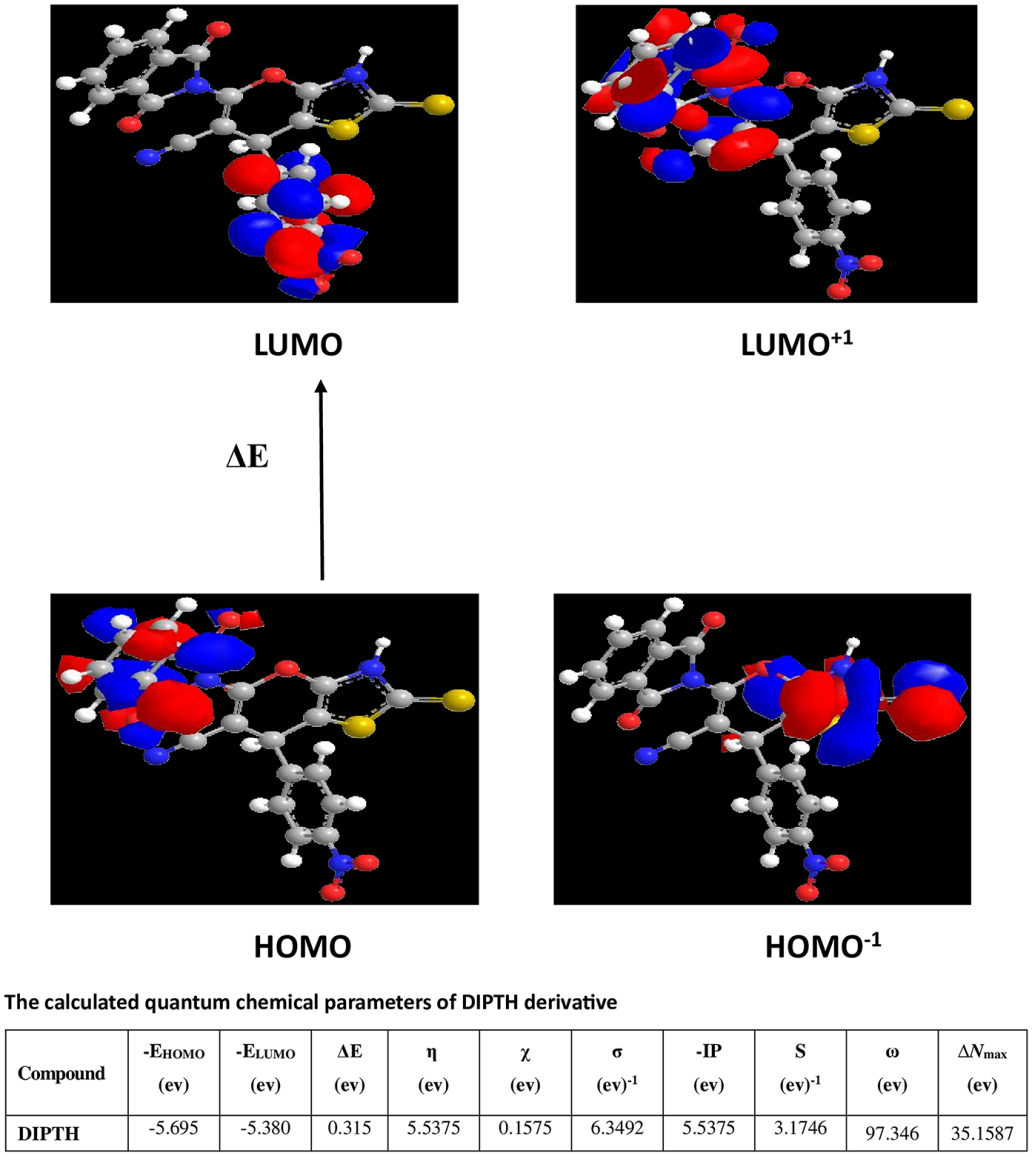
The molecular structures (HOMO & LUMO) as well as the calculated quantum chemical parameters of the synthetic compound (DIPTH).

**Fig. 3:**
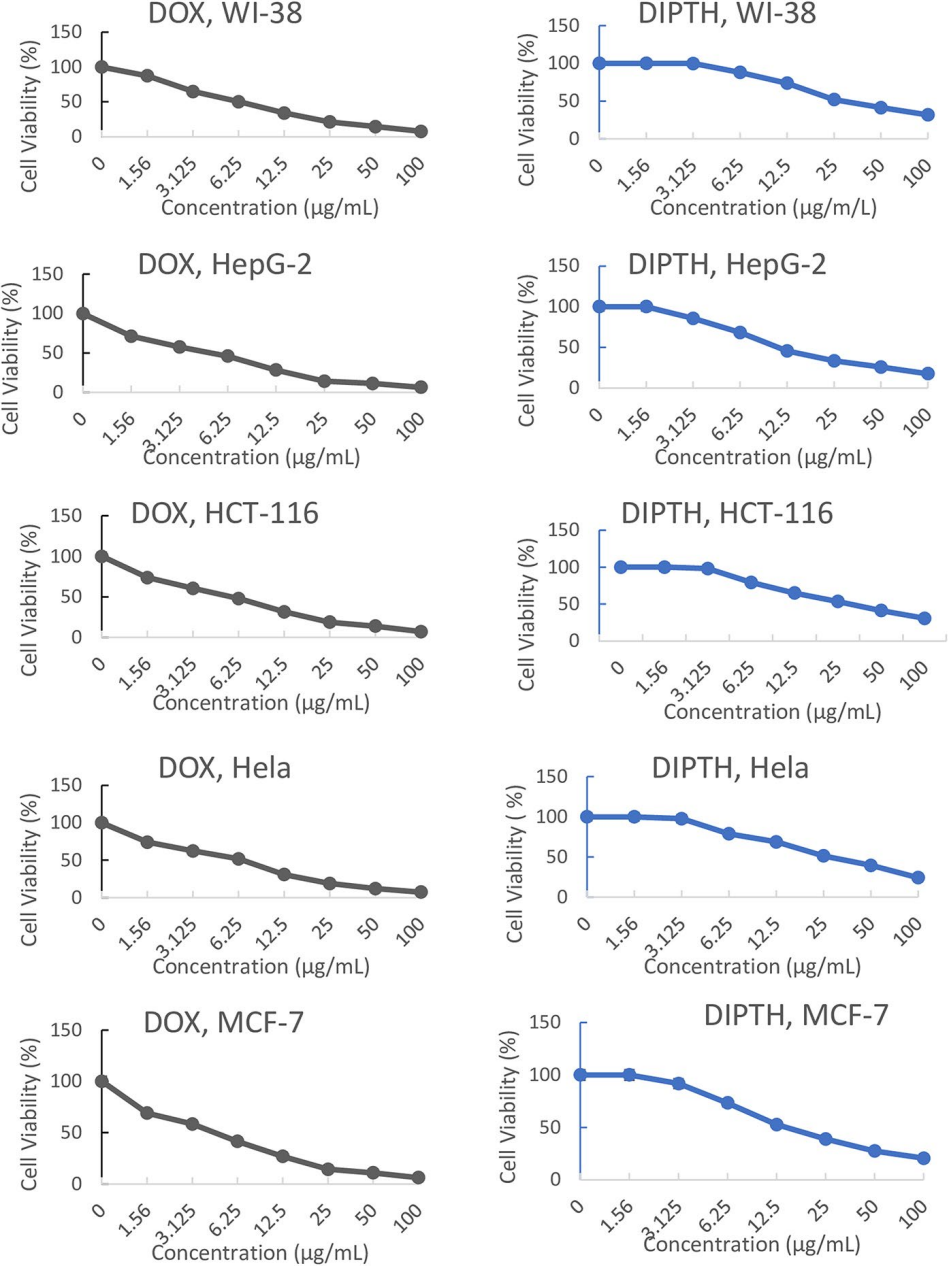
Comparative anticancer activities (in terms of % cell viability) of different concentrations (µg/mL) of doxorubicin (DOX) and the synthesized compound (DIPTH) on WI-38, HepG-2, HCT-116, Hela, and MCF-7.

**Fig. 4:**
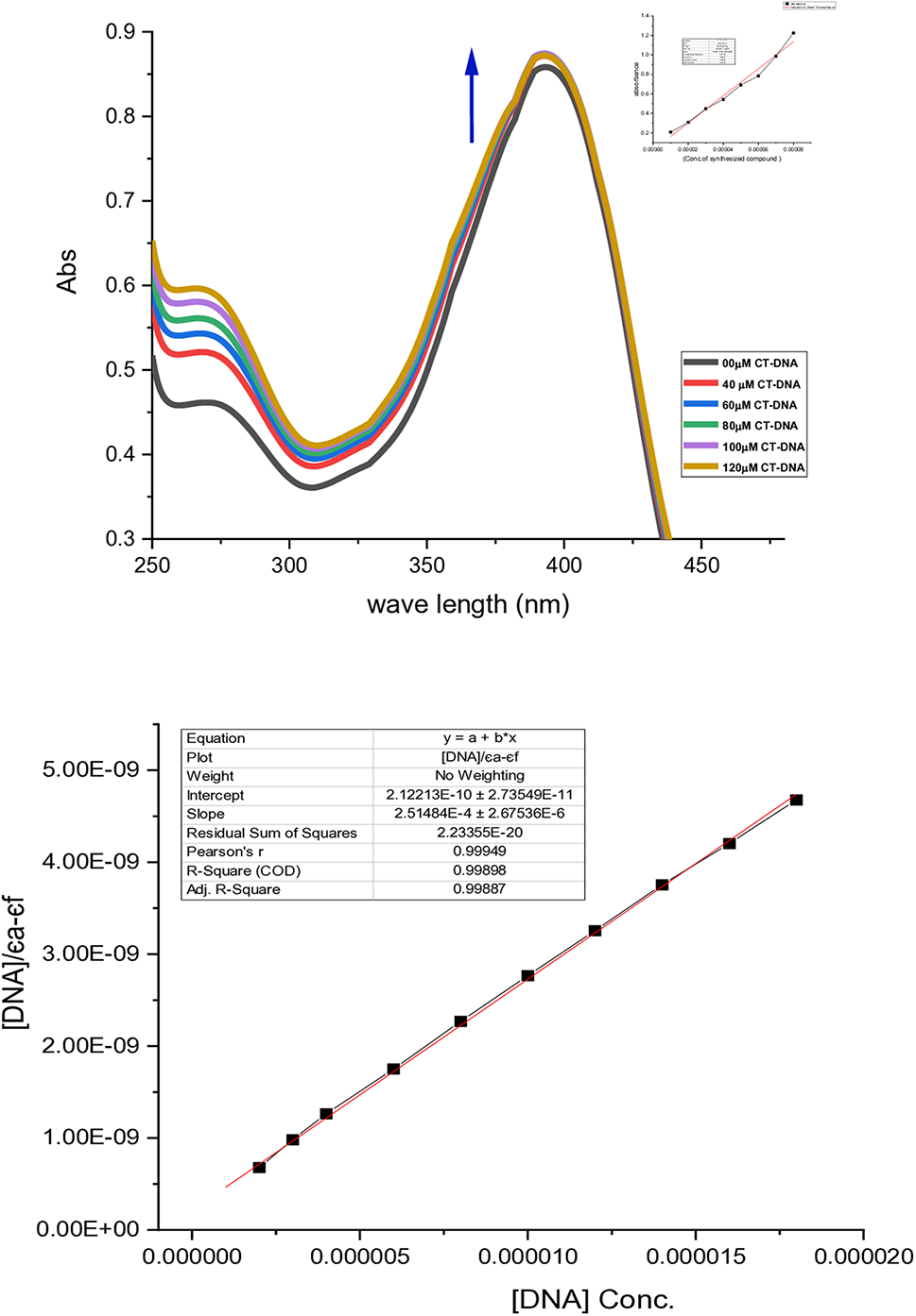
Absorption spectra of the synthesized compound (DIPTH) in buffer pH 7.2 at 25°C in the absence and presence of increasing amount of CT-DNA, Inset: The linear plot for the calculation of the molar extinction coefficient of DIPTH (**Upper panel**) as well as the plot of [DNA]/(εa-εf) versus [DNA] for titration of DNA with the compound (**Lower panel**).

**Fig. 5:**
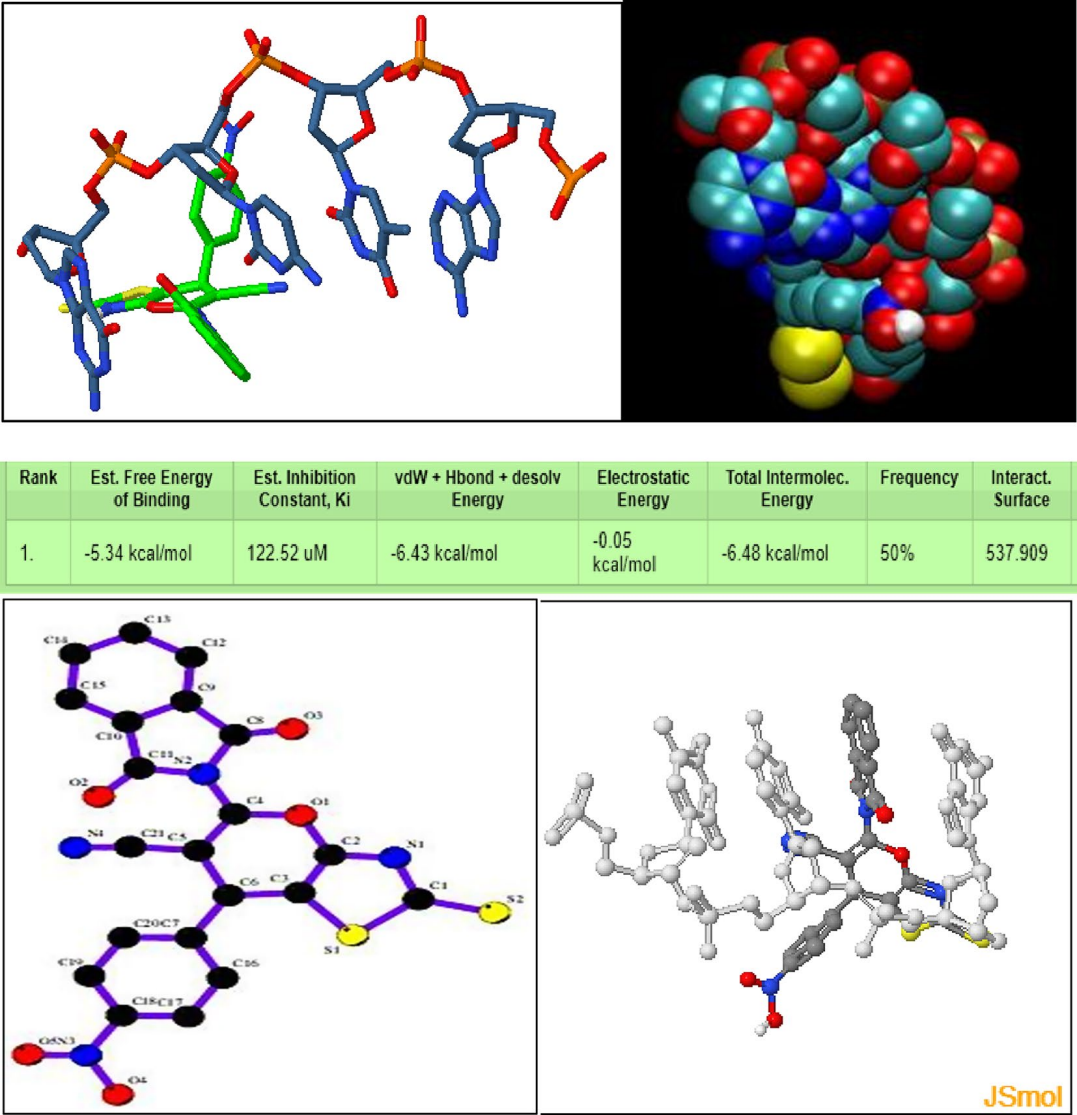
Groove binding to the minor groove of DNA and the intercalation into DNA.

In the current study, initially 4H-pyran template was prepared, using one-pot green reaction. As depicted in Fig. 1, the synthesis of 5-(1,3-dioxoisoindolin-2-yl)-7-(4-nitrophenyl)-2-thioxo-3,7-dihydro-2H-pyrano[2,3-d] thiazole-6-carbonitrile (DIPTH) derivative was efficiently achieved via molecular hybridization reaction of thiazolopyran with isobenzofuran-1,3-dione, according to a one-step synthesis reaction in the absence of solvent. The structure of the synthesized compound, shown in Fig. 1, is confirmed by FT-IR (Fig. 1S), ^1^H-NMR (Fig. 2S), ^13^C NMR (Fig. 3S), MS (Fig. 4S), and UV-Vis spectra (Fig. 5S). The IR spectrum of the compound (DIPTH) showed disappearance of NH_2_ absorption band and exhibited a sharp absorption band at 1709 cm^− 1^ corresponding to C = O beside the presence of absorption band at 2155 cm^− 1^ for C ≡ N. The ^1^H NMR spectrum showed a signal at δ = 3.94 ppm H of CH proton.

### Molecular structure

The newly synthesized compound (DIPTH) molecular structure was optimized using the HF method, and the molecule was constructed with the Perkin Elmer ChemBioDraw and then optimized utilizing Perkin Elmer ChemBio3D software. The molecular orbital pictures of HOMO^− 1^, HOMO, LUMO and LUMO^+ 1^ of DIPTH are given in Fig. 2. The HOMO–LUMO energy gap ‘’ΔE’’ is a key stability indicator used to develop theoretical models for explaining molecular structure and conformation barriers in multiple molecular systems. The smaller the ΔE value, the more stable the compound. Our data demonstrated that the energy gap between the HOMO and LUMO of DIPTH is 0.3I5 eV. The estimated quantum chemical markers are given in Fig. 2. Additional parameters such as electronegativity, η, chemical potentials, IP, hardness, χ, absolute softness, σ, global electrophilicity, ω, global softness, S, and additional electronic charge, ΔN_max_, have been calculated according to the reported equations^56–60^. Soft molecules are of higher reactivity than hard molecules as they can more easily donate electrons to an acceptor. In the study published by Assad et al. (2022), a group of 5 thiazole derivatives showed the following: ΔE in the range from 3.5712 to 6.0298 eV, softness from 0.3317 to 0.56 eV^− 1^, hardness from 3.5003 to 4.612 eV, ionization potential from 6.2806 to 7.1917 eV, and electronegativity from 1.7856 to 3.0149 eV compared to 0.315 eV, 6.3492 eV^− 1^, 0.1575 eV, 5.5375 eV, and 5.5375 eV, respectively showed for our synthesized compound. Further in their study, the compound phthalylsulfathaizole showed the lowest ΔE (3.5712 eV), which is higher than that of our compound (0.315 eV), and the highest softness value (0.5600 eV^− 1^), which is lower than that of our compound (6.3492 eV^− 1^). Depending on their quantum results, they concluded that among the five compounds phthalylsulfathaizole has the highest tendency to function as anticorrosive^61^. In the Ashour et al. (2023) study^62^, 7 thiazole derivatives were investigated as potential anticancer agents. The E_HOMO_ values were in the range of − 5.45 to − 5.86 eV, which is comparable to that of our compound, the E_LUMO_ from − 3.09 to − 4.01 eV, which is lower than that of our compound, ΔE from 1.51 to 2.42 eV, which is higher than that of our compound, hardness from 0.75 to 1.21 eV, which is higher than that of our compound, softness from 0.83 to 1.33 eV, which is lower than that of our compound, and electrophilicity from 7.72 to 15.03 eV, which is lower than that of our compound. They concluded that the 7 compounds showed a small and close energy gap, compound 6 displayed the smallest hardness and highest softness values, and thus it is the most reactive, least stable kinetically, and the most soft. Additionally, all seven compounds were identified as strong electrophiles, as they have an electrophilicity index > 1.5 eV. Our obtained results reveal that chemical hardness and ΔE values of the synthesized compound DIPTH are low, and the absolute softness value is high confirming that our compound is reactive. Further, in light of all the aforementioned quantum-chemical properties, the DIPTH compound is more stable, polarizable, and more reactive, and has a greater tendency to bind with DNA. Thus, there is flexibility in its use for biological purposes^63,64^.

### Antiproliferative activity

The inhibitory activity of the DIPTH at levels of 1.56, 3.125, 6.25, 12.5, 25, 50, and 100 µg/mL was examined by conducting the MTT test on four cancer cell lines (HepG-2, HCT-116, MCF-7, and Hela) and a normal cell line (WI-38). Doxorubicin (DOX) was used as a reference drug. The viability of cells (%) was determined, and the inhibitory concentration (IC_50_) was calculated. The lower the IC_50_ the stronger the antiproliferative activity. Moreover, the selectivity index was calculated using the available normal cell line (WI-38) as (IC_50_ of WI-38/IC_50_ of the respective cancer cell line) to assess the compound’s ability to select between cancer cells and normal cells. If the selectivity index value of a compound is less than 2.0, the compound may have strong anticancer activity but could also be a general toxin^65^. A higher selectivity index value indicates a greater ability of the drug to target cancer cells and, hence, safer for the surrounding non-cancer (normal) cells^66^. Our in vitro cytotoxicity study results are presented in Table 1; Fig. 3. We found that by increasing the concentration of DOX or DIPTH there was a decrease in the percent of the viable cells with the five studied cell lines (WI-38, HepG-2, HCT-116, MCF-7, and Hela) as depicted in Fig. 3 indicating a dose-dependent inhibitory activity on their cellular proliferation. The data presented in Table 1 demonstrates that the newly synthesized compound exhibits strong-to-moderate antiproliferative activity against the five tested cell lines. Specifically, it displayed IC_50_ values of 14.05, 17.77, 29.65, 32.68, and 36.17 µg/mL for HepG-2, MCF-7, Hela, HCT-116, and WI-38, respectively compared to 4.50, 4.17, 5.57, 5.23, and 6.72 µg/mL, respectively for DOX. This suggests that the compound is most effective against liver (HepG-2) and breast (MCF-7) cancer cells while less effective against Hela, HCT-116, and WI-38. Further, compared to the reference drug (DOX) the compound showed much lower toxicity towards normal cells (WI38) [IC_50_ = 36.17 µg/mL for the compound versus IC_50_ = 6.72 µg/mL for DOX, *P* < 0.0001]. This means that our compound was more safe than DOX on the WI38 cell line by 5.38-fold. Therefore, our compound is expected to be safer than DOX towards non-cancerous cells. Besides, the DIPTH showed the highest selectivity index value at 2.57 with HepG-2 cells, compared to 1.49 for DOX, and 2.04 with MCF-7 cells, compared to 1.61 for DOX. However, it also showed selectivity index values less than 2.0 for Hela (1.22) and HCT-116 (1.11), compared to 1.21 for Hela and 1.28 for HCT-116 with DOX. Other researchers have reported selectivity index values below 2.0 for DOX, for example: 0.57 for MCF-7 and 1.31 for Hela^67^, 0.82 for HepG-2^68^, 1.4 for MCF-7^69^, 0.04 for HepG-2^70^, and 0.29 for MCF-7 and 1.01 for HT29^65^.

**Table 1:** In vitro cytotoxicity of the synthesized compound (DIPTH) against MCF-7, HCT-116, HepG-2, Hela, and WI-38.

Compound	Doxorubicin				
Cell lines	WI-38	HepG-2	HCT-116	Hela	MCF-7
IC_50_(µg/mL)	6.72 ± 0.5	4.50 ± 0.2	5.23 ± 0.3	5.57 ± 0.4	4.17 ± 0.2
Selectivity index	1	1.49	1.28	1.21	1.61

Compound	**DIPTH**				
Cell lines	***WI-38***	***HepG-2***	***HCT-116***	***Hela***	***MCF-7***
IC_50_(µg/mL)	36.17 ± 2.3	14.05 ± 1.2	32.68 ± 2.0	29.65 ± 1.8	17.77 ± 1.3
Selectivity index	1	2.57	1.11	1.22	2.04
P value	< 0.0001	0.0002	0.0016	< 0.0001	< 0.0001

^IC^_50_: ^1 – 20 (strong), 21 – 50 (moderate), 51 – 100 (weak), and above 100 (non−cytotoxic)^.

^DIPTH : 5−(1,3−dioxoisoindolin−2−yl)−7−(4−nitrophenyl)−2−thioxo−3,7−dihydro−2 H−pyrano[2,3−d] thiazole−6−carbonitrile^.

^P value is considered statistically significant at *P*<0.05^.

^Human lung fibroblast cell line (WI−38)^.

^Human hepatocellular carcinoma cell line (HepG−2)^.

^Human colorectal carcinoma cell line (HCT−116)^.

^Epitheloid cervix carcinoma cell line (Hela)^.

^Human breast adenocarcinoma cell line (MCF−7)^.

Herein, we can conclude that the DIPTH has a strong toxic activity with high selectivity on the liver cancer (HepG-2) and breast cancer (MCF-7) cells while displaying moderate cytotoxic activity with low selectivity on colorectal cancer (HCT-116) and cervical cancer (Hela) cells.

According to the literature, thiazole derivatives act as anticancers via various mechanisms. They can induce apoptosis, and inhibit tubulin, NFkB/mTOR/PI3K/AkT, and topoisomerase. In addition, they can regulate estrogen-mediated activity^71^.

### DNA binding

Electronic absorption spectroscopy is reported to be an effective method in examining the binding modes and binding extent of DNA with the synthesized compounds. The absorbance spectra of DNA can exhibit hypochromism (decreased absorbance intensity) and hyperchromism (increased absorbance intensity) when titrated with different molecules, indicating interactions between the molecules and the DNA. Hypochromism occurs when a molecule binds to DNA, stabilizing the helix by inserting flat aromatic species between the base pairs. Hyperchromism occurs when the secondary structure of DNA is disrupted^72^.

**Fig. 6:**
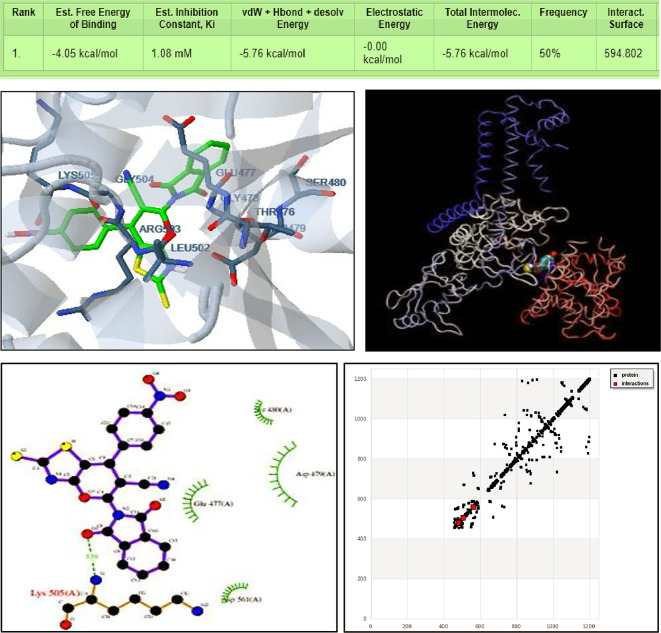
3D representation of the interaction of the synthesized compound (DIPTH) with the active site residues of topoisomerase IIβ, 3QX3 (**Upper panel**; green color in right blue color in left) as well as 2D binding sites of (DIPTH) with human DNA topoisomerase IIβ, 3QX3 (left) and H.B. interaction (right) (**Lower panel**).

In the current study, the absorbance of the compound was firstly recorded by various concentrations, plotted vs. concentration (Fig. 6S) and then the molar extinction coefficient (є_f_) was evaluated from the slope value (13,906 M^− 1^ cm^− 1^). Then, quantification of CT-DNA binding affinity with the synthesized compound (DIPTH) was studied using the electronic spectral technique by measuring the change in absorbance and shift in wavelength upon increasing concentrations of CT-DNA solution in a fixed concentration of DIPTH under physiological pH (7.2) and temperature (25 °C). We have determined the intrinsic binding constant (*K*b) to CT-DNA of the synthesized compound by monitoring the absorption intensity of the charge transfer spectral bands at (λ393 nm). Figure 4 indicates the electronic absorption spectra for DIPTH with and without the addition of CT-DNA. It shows a hyperchromic effect. Hyperchromic effect primarily appears due to alterations in DNA conformation and structure depending on the interaction of compounds to DNA^73^. Herein, the observed hyperchromic effect indicates modifications in DNA architecture after DIPTH-DNA binding resulting in damage to the DNA double-helix structure. The value of *K*b was calculated using the plot of [DNA]/(εa-εf ) versus [DNA] (Fig. 4). The higher the value of *K*b indicates more favorable conditions for the interaction. The calculated *K*b value of the compound is 2.48 × 10^6^ M^− 1^. This *K*b value of DIPTH appears comparable to 4.7 × 10^6^ M^− 1^ for the derivative 4,5,6,7-tetrabromo-2-[3-(4,5,6,7-tetrabromo-1,3-diox-o-2,3-dihydro-1H-isoindol-2-yl)-1H-1,2,4-triazo-l-5-yl]-2,3-dihydro-1H-isoindole-1,3-dione^74^ and to the range of those of some classic DNA-binders, as the Ethidium Bromide complex (*K*b = 1.4 × 10^6^ M^− 1^)^75^ and higher than other classic DNA-binders like doxorubicin (*K*b = 1 to 1.6 × 10^4^ M^− 1^)^76^, methyl-CCNU (semustine) (*K*b = 1 0.53 × 10^3^ M^− 1^), and CCNU (lomustine) (*K*b = 8.12 × 10^3^ M^− 1^)^77^, suggesting that the compound has a strong binding affinity to interactions with the DNA.

## Molecular docking study

Molecular docking is a commonly used technique in drug discovery for the exploration of the binding mode of DNA and the identification of where a compound could bind in the major or minor grooves of DNA^78^.

The docked conformations with the co-crystal structure of DOX-DNA sequence d(CGATCG) complex DNA (PDB ID: 1d12, http://www.rcsb.org/structure/1D12)^79^ was carried out. From the docking study, it is concluded that the synthesized compound is well fitted in the active sites of the DNA and interacts with the favorable binding ability of the major groove of the DNA with binding energy − 5.34 kcal/mol as shown in Fig. 5. In particular, the aromatic moieties of the ligand are found to be extensively involved in π-π stacking non-covalent interactions with the nucleobases of DNA. This result corroborates well with the experimental binding parameters from DNA binding experiment, and clearly explain the potential biological activity of the synthesized compound.

### Molecular docking with human DNA topoisomerase II beta (IIβ)

The interaction between the disoindolinyl pyrano[2,3-d] thiazole derivative (DIPTH) and topoisomerase IIβ (data bank code of 3QX3, http://www.rcsb.org/structure/3QX3) was investigated using molecular docking, The results show an affinity value of a decent binding score of -4.05 kcal/mol and an estimated inhibition constant (Ki) of 2.8 µM. The results indicate the formation of three interactions with three H.B.s in thiazole derivative (DIPTH). The first interaction was assigned between CG, OD1 centers of ASP479, and N1 (3) of DIPTH with a length of 3.08, and 2.75 Å, respectively. The second interaction was found between H1 (33) of DIPTH with OD1 centers of ASP479 with a length of 2.92 Å. The third interaction was observed between H1 (33) of DIPTH with ARG503 (OD1) corresponding to a length of 3.67 Å. The polar interaction was between the center OD1 of ASP479, OE1 of GLN773, and O1 (6) of the ligand with a length of 3.00 Å, and CD, CG center of ARG503 with N1 (3) with a length of 3.42 Å, interaction with hydrogen bonds with LYS505 (-1.2385), ASP479 (CG, OD1) with N1 (3) [2.75 Å], and H1 (33) [2.92 Å], ARG503 (OD1) with H1 (33) [3.67 Å] (Fig. 6).

### Molecular docking with human DNA topoisomerase II alpha (IIα)

**Fig. 7:**
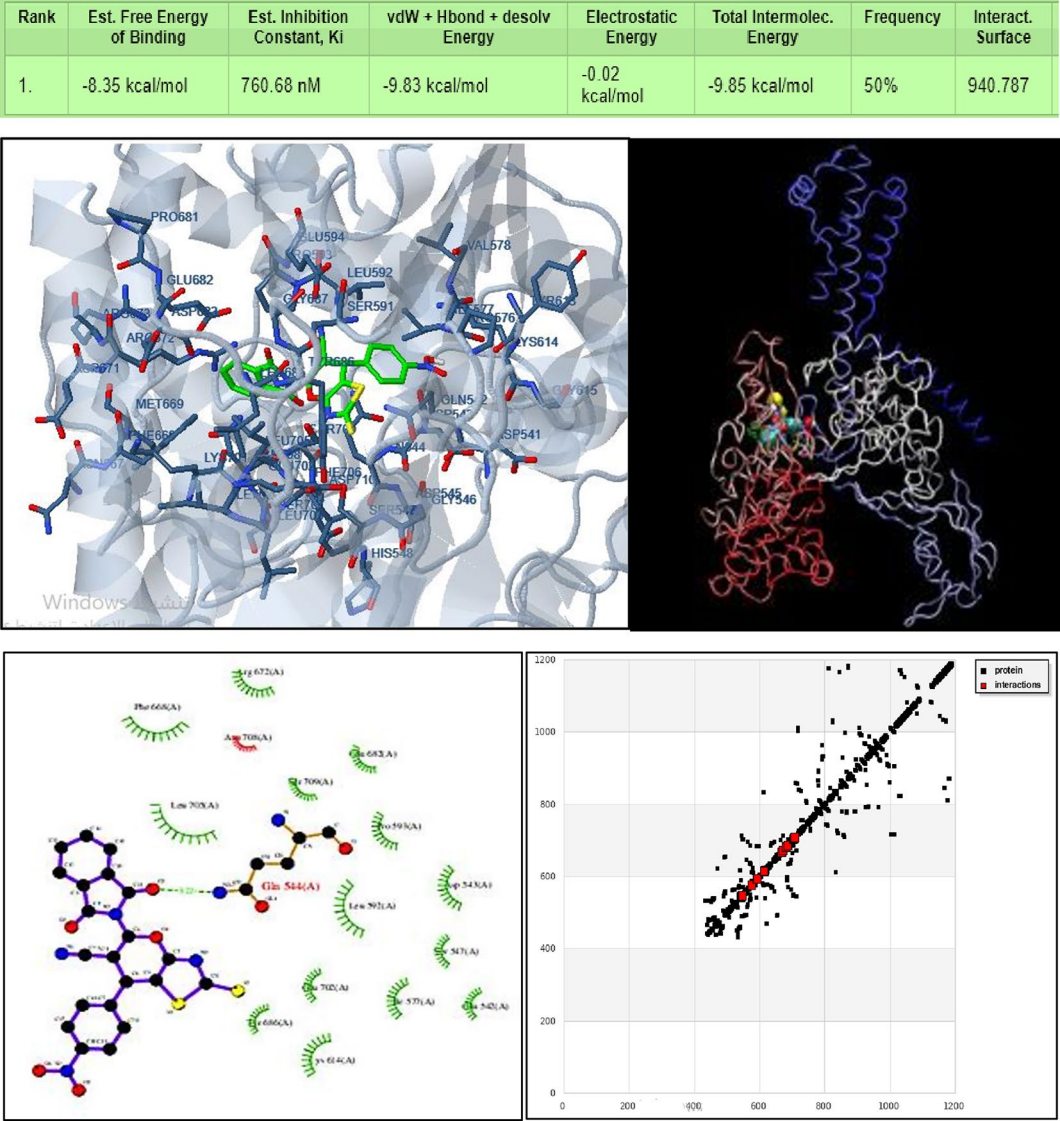
3D representation of the interaction of the synthesized compound (DIPTH) with the active site residues of topoisomerase IIα, 4fm9 (**Upper panel**; green color in right blue color in left) as well as the 2D binding sites of (DIPTH) with human DNA topoisomerase IIα, 4fm9 (left) and H.B. interaction (right) (**Lower panel**).

The results of the molecular docking simulation for compound DIPTH with 4fm9 - ISOMERASE / DNA (http://www.rcsb.org/structure/4FM9) from Fig. 7 where hydrogen bond formed between the center CG, CD, NE2 of GLN544 and O2(21) of the ligand with a length of 3.22 Å, and six polar interactions. The first interaction was between the center OG of SER547 and H1 of the ligand with a length of 3.78 Å. Another interaction site was between NZ of LYS614 and H11, N3 and O4 of the ligand corresponding to a length 3.85, 3.74, 3.64 Å, respectively. A third interaction site was found to be between OG of SER709 and O2 of the ligand corresponding to a length 3.09 Å. A fourth interaction site was assigned between OE2 of GLU682 and O_3_ of the ligand corresponding to a length 3.37 Å. Also pi-pi interaction between CE2,CZ centers of PHE 668 and C13,C14,C15 of the ligand corresponding to a length 3.03, 3.84, 3.30 Å interactions resulting with binding energy − 8.35 kcal/mol.

### Molecular docking with receptor of breast cancer MCF-7

**Fig. 8:**
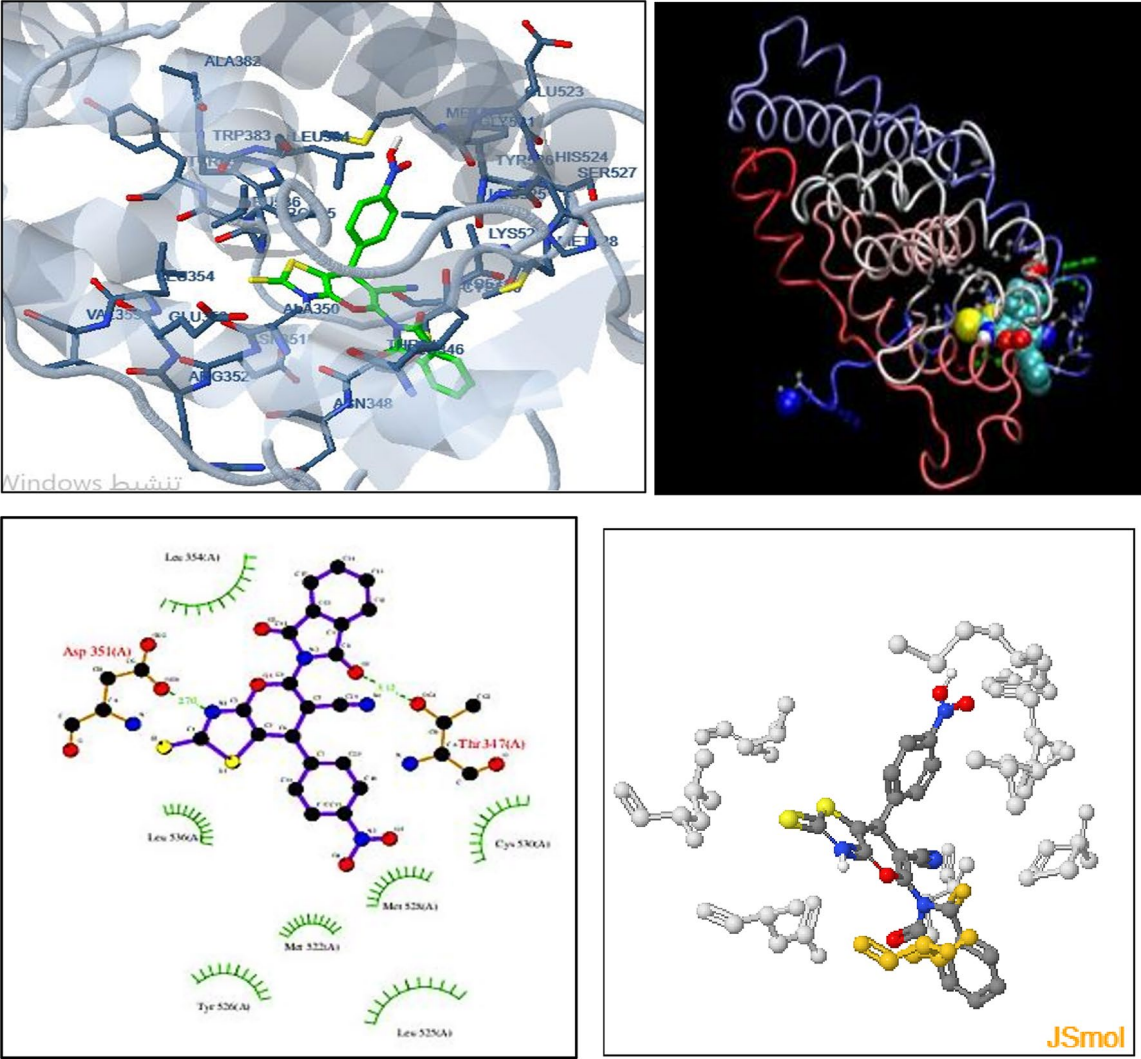
3D representation of the interaction of the synthesized compound (DIPTH) with the active site residues of ERT receptor (**Upper panel**; green color in right blue color in left) as well as the 2D binding sites of (DIPTH) with the active site residues of ERT receptor (left) and H.B. interaction (right) (**Lower panel**).

Human estrogen ER alpha receptor of MCF-7 (PDB ID: 3ERT, http://www.rcsb.org/structure/3ERT) which have good resolution about 1.9 Å, co-crystallized ligand (OHT) was selected for docking study with the newly synthesized compound DIPTH. Docking results with 3ERT present in Fig. 8 showed that the synthesized compound exhibits good docking score (-6.83 kcal/mol) which indicating best binding with the receptor. The interaction of synthesized compound with 3-ERT exhibited two HB with THR-347 and ASP-351; cation-pi with TYR-526 residue; three hydrophobic interaction with CE center of MET 528.

## Conclusions

In our continuous search for potential anticancer drugs, the title compound (DIPTH) was synthesized, and characterized by various spectroscopic techniques. The DNA binding mode of the synthesized compound DIPTH with CT-DNA has been evaluated, anticancer activities of the prepared disoindolinyl pyrano[2,3-d] thiazole derivative (DIPTH) have been studied against HCT-116 (human colon carcinoma) HepG-2 (human hepatocellular carcinoma), MCF-7 (human breast carcinoma), Hela (epitheloid cervix carcinoma), and WI-38 (non-tumorigenic human) cell lines for their anticancer potential candidacy, it reveals that DIPTH exhibited strong anticancer activities against HepG-2 and MCF-7. The molecular docking study demonstrated that DIPTH showed the best-docked score with strong to moderate anticancer potency toward all the enzymes used. The molecular modeling study of the synthesized compound shows the ability to bind with the amino acid residues ASP479 and Arg 503 in topoisomerase II enzyme binding site and embed in DNA grooves.

## Methods

### Materials and apparatus

All used chemicals were obtained from Sigma, Aldrich, Fluka, and Merck. They were not further purified before being used. The melting point was estimated using an electrothermal apparatus and is uncorrected. Elemental analysis was done in the Microanalysis unit at Cairo University. FTIR spectral data were obtained using KBr discs within the range of 4000 –400 cm^− 1^ on a Jasco FTIR-4100 spectrophotometer with a maximum resolution of 0.9 cm^− 1^. UV-Vis spectra: Acquired using a Perkin-Elmer AA800 spectrophotometer Model AAS, with a 1.0 cm cell. A JEOL-ECA 500 II NMR Spectrometer operating at 500 MHz, with DMSO-d6 as the solvent was used for^1^H NMR and^13^C NMR spectra. The chemical shifts are introduced in ppm, with tetramethyl silane (TMS) as the internal reference. A Shimadzu GCMS-QP 1000 EX mass spectrometer, at 70 eV, was used to obtain Mass spectra. Molecular docking investigations were done using MOE (2014.0901) (Molecular Operating Environment Software). The compound was constructed using PerkinElmer ChemBio Draw and optimized with PerkinElmer ChemBio3D software. Docking simulation was accomplished using AutoDock tools. The molecular docking studies were obtained using the three-dimensional X-ray structure of protein (3QX3), the co-crystal structures of human topoisomerase II beta in complex with DNA & etoposide proteins and (1D12-DNA) a beta DNA dodecamer conformation & dynamic, respectively, using MOE. The selected enzymes were enclosed in a box with number of grid points in x × y × z directions, 20 × 20 × 20.

### Synthesis of 5-(1,3-dioxoisoindolin-2-yl)-7-(4-nitrophenyl)-2-thioxo-3,7-dihydro-2 H-pyrano[2,3-d]thiazole-6-carbonitrile (DIPTH)

In 10 mL acetic acid, a mixture of 5-amino − 7-(4-nitrophenyl)-2-thioxo-3,7-dihydro2H-pyrano[2,3-d]thiazole-6-carbonitrile (PTH**)** (1.66 g, 5 mmol) and isobenzofuran-1,3-dione (0.74 g, 5 mmol) was heated for 2 h under reflux. The solid was gathered and crystallized from ethanol to give brownish orange crystals; yield (85%); m.p.: 208–211 °C; IR (KBr, cm^− 1^): 3076 (C–H aromatic), 2846.9 (C–H), 2214 (C ≡ N), 1709 (C = O), 1617 (C = C);^1^H-NMR (DMSO-d6)δ: 3.94 (s, 1 H, CH), 6.70– 6.73 (d, 2 H, J = 8 Hz, Ar–H), 7.52 (d, 2 H, Ar–H), 7.87 (d, 2 H, J = 8 Hz, Ar–H), 8.13 (d, 2 H,, Ar–H);^13^C-NMR (100 MHz) (DMSO-d_6_) δ: 26.2, 117.4, 125.9, 129.6, 130.1, 131.5, 33.3, 134.6, 165.4, 196.3. (ES- MS, m/z (%): 462.35Anal. Calcd. for C_21_H_10_N_4_O_5_S_2_ (462.44): C, 54.54; H, 2.18; N, 12.12; found: C, 53.54; H, 2.21; N, 12.37.

### Cytotoxicity investigation

#### Cell lines and chemical reagents

Human lung fibroblast (WI-38), human breast adenocarcinoma (MCF-7), colorectal carcinoma (HCT-116), epitheliod cervix carcinoma (Hela), and hepatocellular carcinoma (HepG-2) cell lines were obtained from ATCC via Holding company for biological products and vaccines (VACSERA), Cairo, Egypt.

Doxorubicin (DOX) was acquired from a local pharmacy in Egypt and utilized as a standard anticancer medication for comparison. The reagents RPMI-1640 medium, MTT (3-(4,5-dimethylthiazol-2yl)-2,5-diphenyltetrazoliumbromide), dimethyl sulfoxide (DMSO) (sigma co., St. Louis, USA), and Fetal Bovine serum (GIBCO, UK) were used.

#### Antiproliferative activity (MTT assay)

The (WI-38), (MCF-7), (HCT-116), (Hela), and (HepG-2) cell lines were used to determine the inhibitory effects of the synthesized compound on cell growth using the MTT-based method as previously reported^80,81^. This colorimetric assay relies on the conversion of MTT (yellow tetrazolium bromide) to a purple formazan derivative by mitochondrial succinate dehydrogenase in viable cells. The cells were cultivated in RPMI-1640 medium containing 10% fetal bovine serum, 100 units/mL penicillin, and 100 µg/mL streptomycin, and then incubated at 37 °C in a 5% CO_2_ environment. The cell lines were implanted in a 96-well plate (1.0 × 10^4^ cells/well) at 37 ^o^C for 48 h under 5% CO_2_. Following incubation, the cells were treated with various levels of the tested compound for 48 h, and then 20 µL of MTT (5 mg/mL) was added and incubated for 4 h. DMSO (100 µL) was added into each well to solubilize the formed purple formazan. The absorbance was read at 570 nm with a plate reader (EXL 800, USA). The relative cell viability (%) was determined: (Absorbance of treated samples/Absorbance of untreated sample) X 100.

#### Selectivity index

The selectivity index was assessed by dividing the mean IC_50_ value of the normal cell line by the mean IC_50_ value for a cancer cell line^66^.

### DNA binding studies

The newly synthesized compound tends to interact with DNA experimentally and by docking studies theoretically.

#### Absorption spectral studies

Tris HCl buffer (5 mM tris(hydroxymethyl) aminomethane, pH = 7.2, and 50 mM sodium chloride was utilized as a diluting solution. The stock solution of CT-DNA was prepared by dissolving it in the Tris buffer at physiological conditions (pH 7.2 and 25 °C), then stored at 4 °C and used in no more than four days of preparation. The extinction coefficient (approx. 6,600 M^− 1^ cm^− 1^, at 260 nm) was used to estimate the DNA content in Tris HCl buffer, therefore the absorbance values at 260 and 280 nm of DNA were recorded to emphasize its purity. The A260/A280 ratio is normally 1.8–1.9 for pure DNA that is free of protein^82,83^. Original solutions of the substances under investigation were produced in dimethylformamide and diluted in Tris HCl buffer to the required concentration. The titrations were carried out by keeping the compound concentration constant while increasing the DNA concentration. The addition of the same amount of DNA to both the synthesized compound and reference solution removed the absorbance of DNA. The Intrinsic binding constant (*K*b) was calculated from the ratio of intercept to the slope of a plot of [DNA]/(εa-εf) vs. [DNA] of the Wolfe–Shimmer equation ^84^:

$$\frac{{[{\text{DNA}}]}}{{\varepsilon _{{\text{a}}} - \varepsilon _{{\text{f}}} }} = \frac{{[{\text{DNA}}]}}{{\varepsilon _{{\text{b}}} - \varepsilon _{{\text{f}}} }} + \frac{1}{{{\text{K}}_{{\text{b}}} (\varepsilon _{{\text{b}}} - \varepsilon _{{\text{f}}} )}}$$

Where,

- [DNA] is the concentration of CT-DNA in base pairs.
- є_a_ is the extinction coefficient observed for the complex bound to DNA (A_obs_/[compound]) at the given DNA concentration.
- є_f_ is the extinction coefficient of the free compound in solution.
- є_b_ is the extinction coefficient of the compound when fully bound to DNA.

#### Molecular docking studies

Molecular docking technique can be used as a tool to predict the drug-DNA interactions for the rationale design as well as in the mechanistic study by placing a small molecule into the binding site of the target specific region of the DNA mainly in a non-covalent fashion^85^.

. Molecular docking investigations were carried out using MOE (2014.0901) (Molecular Operating Environment Software). The molecules were assembled with Perkin Elmer ChemBio Draw and then optimized. The 3D structure of the newly synthesized compound was obtained. Similarly, the 3D structures of different used proteins were retrieved from the Worldwide Protein Data Bank (http://www.rcsb.org). The Molecular Docking Server^86^ was used to perform docking calculations. The ligand atoms were given Gasteiger partial charges. Rotatable bonds were defined after nonpolar hydrogen atoms were combined. The fundamental hydrogen atoms, Kollman unified atom type charges, and solvation parameters were all added using Auto Dock tools^87^. The Lamarckian genetic algorithm (LGA) and the Solis & Wets local search approach were used to simulate docking^88^. Each docking experiment was built from two separate runs, each of which was limited to 250,000 energy evaluations. A population of 150 was set. A translational step of 0.2 was used during the search, as well as quaternion and torsion steps of 5^89^.

#### Statistical analysis

Data were statistically analyzed by Instat software, version 3.10 (GraphPad, Inc., Sorrento Valley, San Diego, CA, USA) and presented as the means ± standard deviation (SD). Student’s t-test was employed to compare the means between two groups. *P* < 0.05 was considered significant.

## Electronic supplementary material

Below is the link to the electronic supplementary material.

Supplementary Material 1

## Data Availability

The datasets generated during and/or analyzed during the current study are available in the Worldwide Protein Data Bank (wwPDB) repository (http://www.rcsb.org), http://www.rcsb.org/structure/1D12, http://www.rcsb.org/structure/3QX3, http://www.rcsb.org/structure/4FM9, and http://www.rcsb.org/structure/3ERT.
