# Supplementary material for: New thiazole derivative as a potential anticancer and topoisomerase II inhibitor
Source: Sci Rep. 2025 Jan 3;15:710. doi: 10.1038/s41598-024-81294-1 (PMC11698983; doi:10.1038/s41598-024-81294-1)
Supplement: Supplementary file 1 — Supplementary Material 1 [file 41598_2024_81294_MOESM1_ESM.docx]

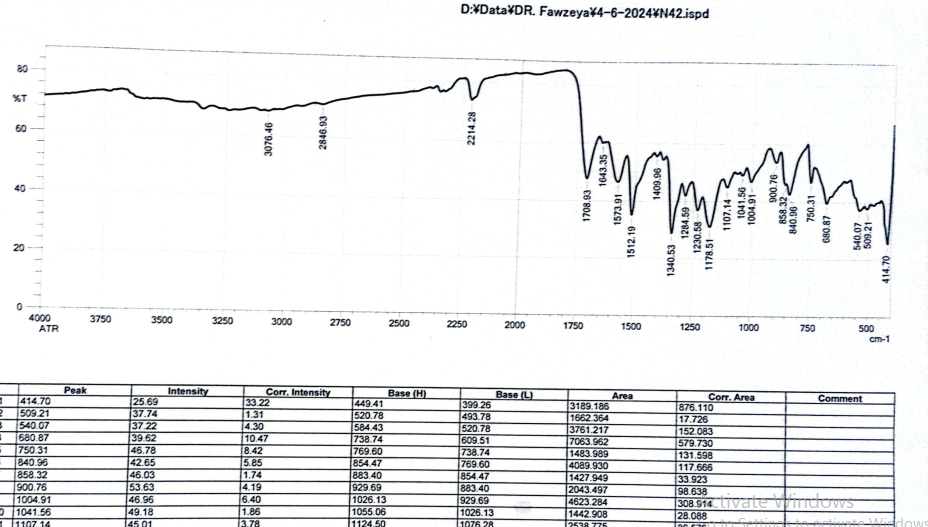


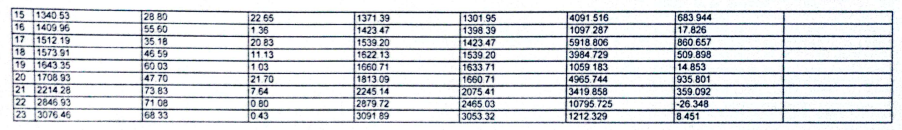


**Fig. 1S.** IR spectrum of the compound DIPTH.


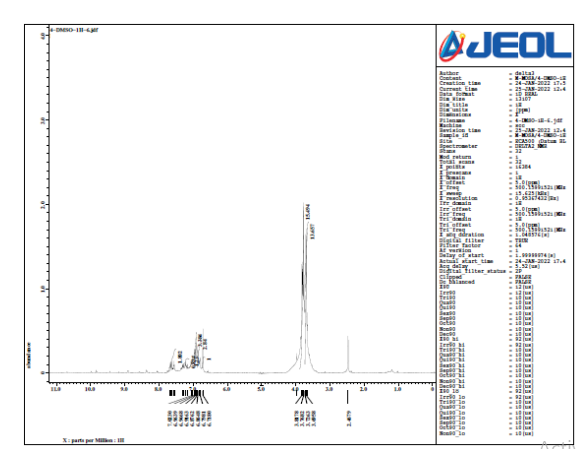


**Fig. 2S.** ^1^H-NMR spectrum of the compound DIPTH.


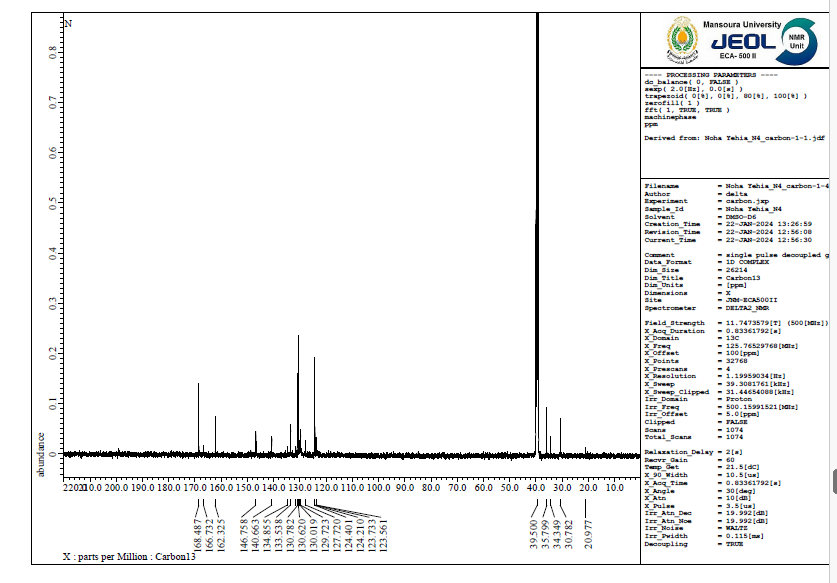


**Fig. 3S.** ^13^C NMR spectrum of the compound DIPTH.


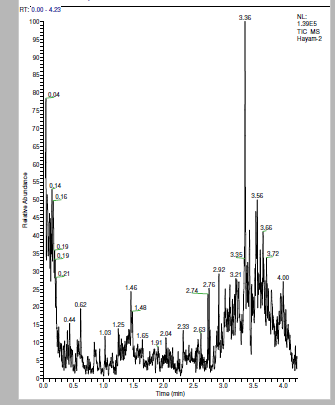


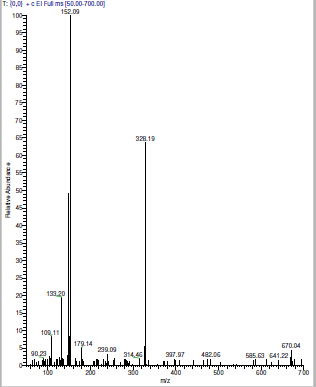


**Fig. 4S.** Mass spectrum of the compound DIPTH.

**Fig. 5S.** UV-Vis spectrum of the compound DIPTH.

**Fig. 6S.** The linear plot for the calculation of the molar extinction coefficient of the compound DIPTH.
